# BEHAVIORAL HEALTH WORKFORCE DEVELOPMENT TO IMPROVE DEMENTIA CARE IN INTEGRATED PRIMARY CARE

**DOI:** 10.1093/geroni/igz038.1391

**Published:** 2019-11-08

**Authors:** Laura O Wray, Bonnie M Vest, Laura Levon Brady, Paul R King

**Affiliations:** 1 VA Center for Integrated Healthcare, Buffalo NY, United States; 2 University at Buffalo, jacobs School of medicine and biomedical sciences, Buffalo, New York, United States; 3 VA Center for Integrated Healthcare, Buffalo, New York, United States

## Abstract

People with dementia (PwD) receive most of their health care in primary care, yet timely recognition and optimal management of dementia in that setting continues to be challenging. Implementation of primary care medical home models in the Veterans Health Administration (VHA) holds promise for improving quality and coordination of dementia care through interprofessional collaboration. Integrating behavioral health providers (BHPs) into primary care may help to support the care of people with dementia and their families. However, most integrated BHPs have a generalist training background and likely require professional education to address the unique needs of patients with dementia. We will describe findings from a national VHA education needs survey of integrated BHPs and an in-depth qualitative study examining primary care for PwD in two large VHA healthcare systems. We will discuss how geriatric experts can serve as trainers to address current gaps in primary care of PwD.

